# Instance Segmentation and Ensemble Learning for Automatic Temperature Detection in Multiparous Sows

**DOI:** 10.3390/s23229128

**Published:** 2023-11-12

**Authors:** Hongxiang Xue, Mingxia Shen, Yuwen Sun, Haonan Tian, Zihao Liu, Jinxin Chen, Peiquan Xu

**Affiliations:** 1 College of Engineering, Nanjing Agricultural University, Nanjing 210031, China; 2020212014@stu.njau.edu.cn (H.X.); 9203020113@stu.njau.edu.cn (Z.L.); chenjinxin@stu.njau.edu.cn (J.C.); 2 Key Laboratory of Breeding Equipment, Ministry of Agriculture and Rural Affairs, Nanjing 210031, China; mingxia@njau.edu.cn (M.S.); 9203021221@stu.njau.edu.cn (H.T.); 2022219003@stu.njau.edu.cn (P.X.); 3 College of Artificial Intelligence, Nanjing Agricultural University, Nanjing 210031, China

**Keywords:** sow, temperature monitoring, instance segmentation, ensemble learning

## Abstract

The core body temperature serves as a pivotal physiological metric indicative of sow health, with rectal thermometry prevailing as a prevalent method for estimating core body temperature within sow farms. Nonetheless, employing contact thermometers for rectal temperature measurement proves to be time-intensive, labor-demanding, and hygienically suboptimal. Addressing the issues of minimal automation and temperature measurement accuracy in sow temperature monitoring, this study introduces an automatic temperature monitoring method for sows, utilizing a segmentation network amalgamating YOLOv5s and DeepLabv3+, complemented by an adaptive genetic algorithm-random forest (AGA-RF) regression algorithm. In developing the sow vulva segmenter, YOLOv5s was synergized with DeepLabv3+, and the CBAM attention mechanism and MobileNetv2 network were incorporated to ensure precise localization and expedited segmentation of the vulva region. Within the temperature prediction module, an optimized regression algorithm derived from the random forest algorithm facilitated the construction of a temperature inversion model, predicated upon environmental parameters and vulva temperature, for the rectal temperature prediction in sows. Testing revealed that vulvar segmentation IoU was 91.50%, while the predicted MSE, MAE, and R^2^ for rectal temperature were 0.114 °C, 0.191 °C, and 0.845, respectively. The automatic sow temperature monitoring method proposed herein demonstrates substantial reliability and practicality, facilitating an autonomous sow temperature monitoring.

## 1. Introduction

The sow, pivotal in contemporary large-scale swine production, propels farm production. Sow health conditions exhibit a direct correlation with its reproductive performance. Temperature emerges as a crucial indicator of a sow’s health status, physiological condition, and environmental adaptability. Based on previous studies, manually collecting temperatures from 1000 sows requires 44 h, which mainly includes thermometer disinfection, measurement operation, temperature reading, data recording, and data statistics [1]. Under the framework of modern intensive farming practices, manual monitoring of sow temperature no longer aligns with the requisites of proficient sow management.

In light of the rapid advancement of automated temperature measurement technology, infrared thermography (IRT) imaging technology is progressively applied in large-scale sow management. Currently, researchers have embarked on studies related to sow lameness detection, farrowing prediction, febrile pig detection, metabolic function assessment in pigs, and early shoulder sore identification in sows, utilizing IRT imaging technology [2,3,4,5,6].

Although infrared thermography (IRT) imaging technology facilitates the rapid acquisition of sows’ surface temperatures, its efficacy is compromised by factors including measuring distance, the quantity of body hair at the measurement site, thermal windows (such as the ear root, eyes, and vulva), and environmental variables (such as temperature, humidity, and wind speed). Consequently, the surface temperature data directly obtained via infrared thermography may not accurately reflect the physiological states of the sows. To address this issue, Jia et al. collected surface temperatures from various sow parts (eyes, ear root, back, and vulva) and rectal temperature, alongside environmental parameters, determining that the surface temperatures of the vulva and the ear root are comparatively less influenced by environmental factors, thereby constituting a robust thermal window for sow temperature monitoring [7]. Nevertheless, an automatic image acquisition of the sows’ ear root area proves challenging due to the animals’ head movement, postural changes, and the limited size of the ear root area.

Moreover, in the predominant body of existing studies utilizing IRT imaging technology for acquiring the surface temperature of sows, temperature data have been obtained by manually extracting the thermal window region, delineating a method that is notably time-intensive. In an endeavor to address this predicament, several scholars have combined target detection algorithms and morphological methods to facilitate thermal window region segmentation. Nonetheless, this strategy necessitates the manual design of feature extractors, thereby posing challenges in managing varying scenarios and sow postures [8,9]. In the pursuit of enhancing the speed and accuracy of sow temperature monitoring, this study proposes a sow temperature monitoring method based on instance segmentation and ensemble learning.

The present study delineates the following principal contributions:

(1) Regarding the segmentation of the thermal window region, an instance segmentation algorithm, predicated on YOLOv5s and DeepLabv3+, is proposed, incorporating the Convolutional Block Attention Mechanism (CBAM) and the MobileNetv2 network to facilitate the precise localization and rapid segmentation of the vulva region.
(2) Pertaining to temperature prediction, a random forest algorithm, optimized by an adaptive genetic algorithm, constructs an inversion model based on environmental parameters, vulvar temperature, and rectal temperature, aspiring to predict the sows’ temperature accurately.
(3) In the context of practical farming, test results exhibit that the sow temperature monitoring method posited in this study can accurately predict the rectal temperature of sows in sheltered scenarios, thereby establishing a foundation for health monitoring and estrus detection in sows.

Subsequent sections of the paper are organized as follows: Section 2 delineates data collection, dataset construction, and model construction; Section 3 proffers an analysis of the results; Section 4 provides the discussion; and Section 5 furnishes the conclusion.

## 2. Materials and Methods

### 2.1. Overall Workflow

**Figure 1:**
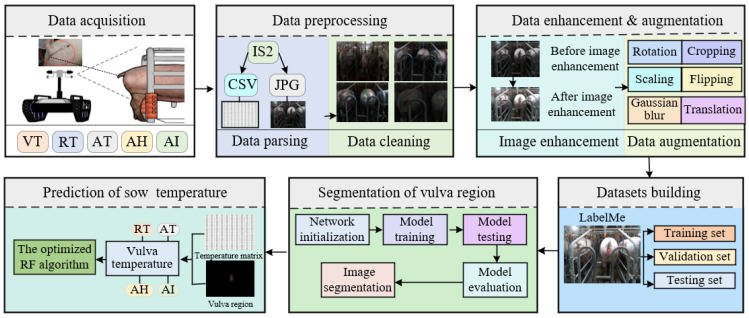
Overall flow chart.

The study methodology is illustrated in Figure 1. Initially, the data acquisition system acquired IR image data from the sows’ and environmental data from the collection location. Subsequently, these raw data were parsed and cleansed to remove outliers. Data augmentation techniques were then applied to generate enhanced raw images. The entire dataset was subsequently divided into training, validation, and testing sets. The constructed instance segmentation algorithm was employed to segment the sows’ vulva region, outputting the coordinate information of this region. These coordinates of the segmented vulva region were then mapped to the corresponding temperature matrix of the thermal infrared image. In the final phase, to ensure the precise prediction of sow temperatures, an enhanced random forest algorithm was implemented to develop a rectal temperature prediction module, relying on environmental parameters and the temperature of the sows’ vulva region.

### 2.2. Animals, Housing, and Data Collection

#### 2.2.1. Experimental Animals, Site, and Time

**Figure 2:**
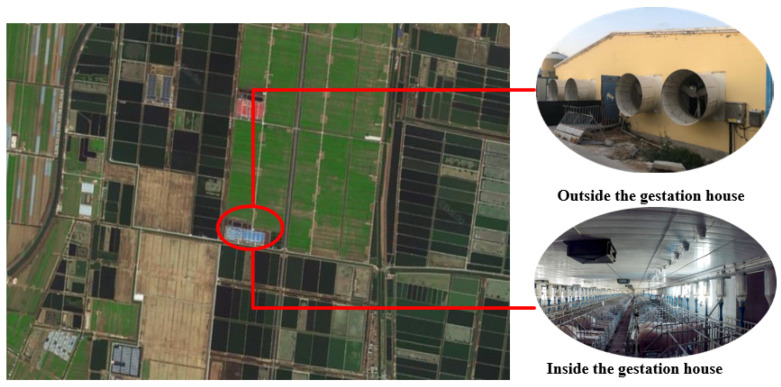
Experimental environment.

Experimental data were procured between 15 September 2021 and 15 March 2022 at Shangbao Herd, Sheyang County, Yancheng City, Jiangsu Province, China. The experimental sow farm is situated in the southeastern coastal region of China, with coordinates spanning 33°31′12″ N to 34°07′15″ N and 119°55′48″ E to 120°34′47″ E. This location lies within a typical subtropical monsoon climate zone. Further geographical and environmental details are presented in Figure 2. The study encompassed 500 cross-bred sows (Large White × Landrace) that were second-parity and multiparous, housed in gestation crates.

#### 2.2.2. Data Acquisition

**Figure 3:**
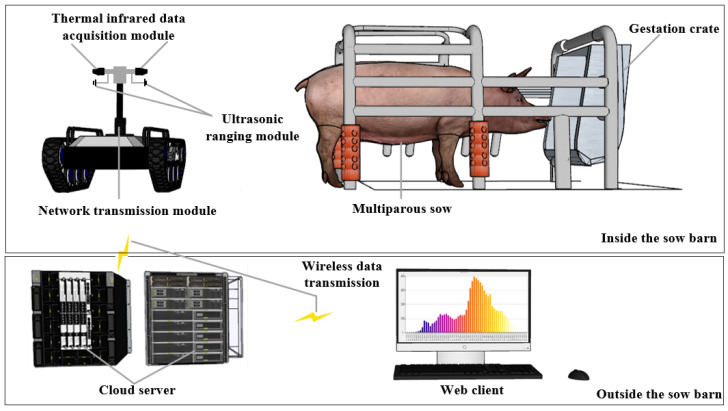
The data acquisition system.

The data acquisition system primarily comprises six components: a mobile robotics platform, an environmental data acquisition module, a thermal infrared data acquisition module, an ultrasonic ranging module, a network transmission module, and a cloud server, as detailed in Figure 3. The TR500S robotic mobile platform, produced by Shenzhenlink Technology Co., Ltd., Shenzhen, China, was employed to transport the individual data acquisition modules and oversee inspection tasks within the gestation crate. Environmental parameters such as air temperature (AT), relative humidity (RH), and illumination (AI) were measured using the TY-DH3 environmental data acquisition module from Tengyu Electronic Technology Co., Ltd., Handan, China. This module boasts an accuracy of ±0.3 °C for temperature, ±3% for RH, and ±5% for illuminance. Sow thermal infrared data were captured using the Fluke Ti401 PRO thermal infrared data acquisition module, a product of Fluke Corporation, Everett, WA, USA, which has a temperature measurement precision of ±2 °C. The robot’s push rod tip features an HC-SR04 ultrasonic ranging module, a creation of Kobee Electronic Technology Co., Ltd., Shenzhen, China, with a 3 mm accuracy, ensuring the thermal infrared data acquisition module maintains a distance of approximately 0.3 m from the sow’s vulva. All acquired data are archived on the cloud server via the network transmission module.

Temperature data of the sows were acquired during two intervals: from 9:00–10:00 a.m. and 2:00–3:00 p.m. The collection process began with the procurement of thermal infrared and environmental data, followed by the measurement of the sows’ rectal temperatures using a mercury thermometer. Throughout the study, a cumulative total of 2900 samples were gathered. Among them, 1–2 data samples were collected for each sow at a time, and temperature data were collected five times for each sow throughout the entire experimental period. All experimental designs and methodologies were sanctioned by the Committee of Animal Research Institute at Nanjing Agricultural University, Nanjing, China.

#### 2.2.3. Data Preprocessing and Dataset Building

The raw thermal infrared data were processed using the SmartView software (version 4.3.0), facilitating the extraction of temperature matrix files, visible light (VL) images, and infrared thermal (IT) images [10]. The VL images provide a more discernible delineation of the sow’s vulva region in comparison to IT images. While aligning and fusing VL and IT images can enhance image quality, this process demands extensive computational resources and compromises the detection’s real-time performance. Consequently, this study employed VL images to establish the instance segmentation dataset. Images that were blurred, those where pens entirely obscured the vulva region, and VL images of sows with high similarity were excluded from consideration.

**Figure 4:**
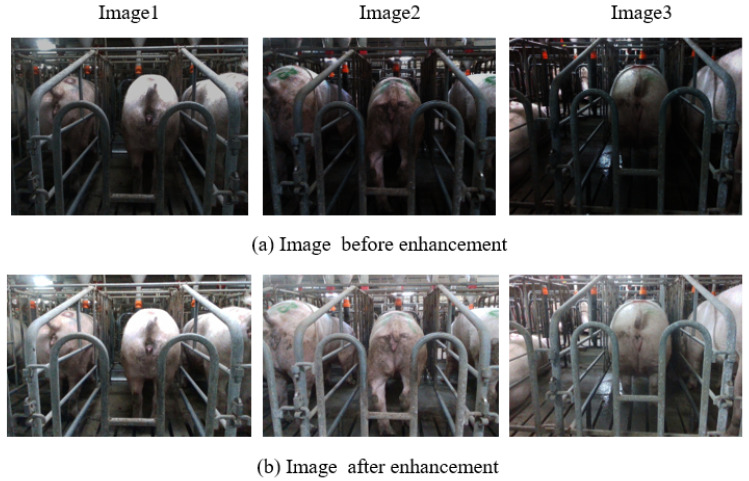
Before and after adaptive histogram equalization. (**a**) Image before enhancement. (**b**) Image after enhancement.

Owing to the suboptimal illumination within the sow barn, some images exhibited reduced light levels. To address this limitation, adaptive histogram equalization was employed to enhance the images, and the processing outcomes are depicted in Figure 4. The initial set of 2850 raw images was expanded to 3200 through data augmentation methods, including rotation, cropping, and scaling. The LabelMe software (version 4.6.0) facilitated the annotation of the sow’s vulva region. Subsequently, the dataset was partitioned into training, validation, and test sets in an 8:1:1 ratio.

### 2.3. Sow Vulva Region Segmentation Model Construction

**Figure 5:**
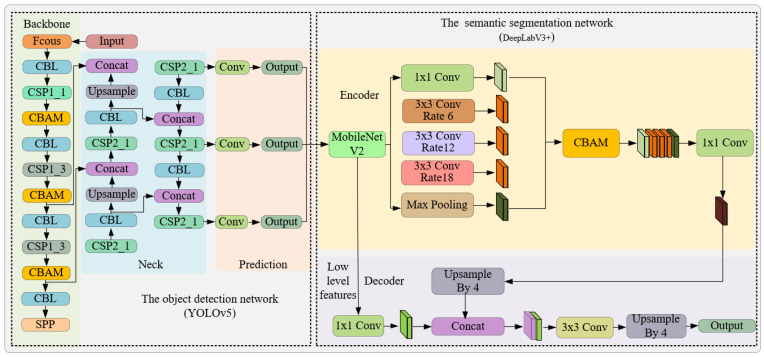
Framework diagram of instance segmentation algorithm based on YOLOv5 and DeepLabv3.

To facilitate swift and precise segmentation of the sow’s vulva region, this study introduces an instance segmentation network amalgamating YOLOv5s and DeepLabv3+. The architecture of this network is depicted in Figure 5. Initially, the enhanced YOLOv5s object detection network is employed to pinpoint the location of the sow’s vulva, thereby mitigating the complexities introduced by varied backgrounds and augmenting segmentation speed. Subsequently, within the delineated bounding box, the refined DeepLabv3+ network undertakes the task of segmenting the vulva region. In an endeavor to direct the model’s attention towards the sow’s vulva region and elevate feature representation, the CBAM module was incorporated into both the YOLOv5s and DeepLabv3+ networks. Conclusively, MobileNetV2, instead of the conventional Xception, was chosen as the backbone network for feature extraction to diminish the model’s complexity and parameter count.

#### 2.3.1. YOLOv5 Network Model

Given the diminutive dimensions of the sow’s vulvar region coupled with a convoluted background, it is imperative to employ an object detection algorithm to pinpoint the sow’s vulvar region prior to its segmentation. Presently, deep learning-based detection algorithms can be bifurcated into two categories: one-stage and two-stage. In pragmatic scenarios, the one-stage algorithms are favored over their two-stage counterparts due to their expedited processing capabilities. Amongst these single-stage detection strategies, YOLOv5 is renowned for its adeptness in harmonizing detection velocity with accuracy and has been widely adopted in the behavioral classification of sows [11]. In this investigation, YOLOv5 is selected as the primary detector for the sow’s vulvar region, further enhanced by the integration of the CBAM module, culminating in the efficacious discernment of the vulvar region.

**Figure 6:**
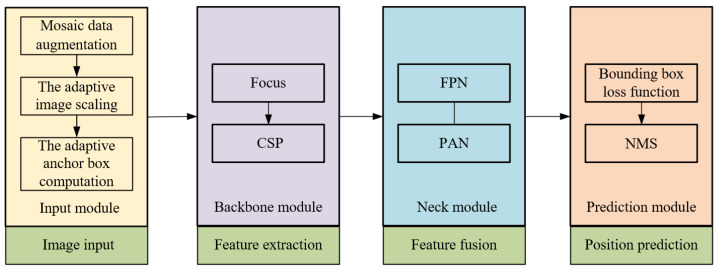
YOLO v5 network model structural sketch.

The YOLOv5 algorithm, a network framework, derives its foundation from YOLOv3-SPP and YOLOv4. This framework encompasses four distinct versions: YOLOv5s, YOLOv5m, YOLOv5l, and YOLOv5x. The YOLOv5 network model is structured into four segments: the input module, backbone module, neck module, and prediction module. Specifically, the input module manages the intake of the acquired VL images. The backbone module focuses on the primary feature extraction of the sow’s vulva region. Meanwhile, the neck module facilitates the fusion of these vulva region features. Finally, the prediction module is dedicated to ascertaining the precise location of the sow’s vulva region. A comprehensive schematic illustrating the YOLOv5 network architecture can be viewed in Figure 6.

The input module utilizes mosaic data augmentation, adaptive image scaling, and adaptive anchor box computation to enhance the richness of background information [12]. The backbone module incorporates primarily the Cross-Stage Partial (CSP) and the Focus modules. Within the backbone module, the CSP aims to amplify the learning efficiency of the convolutional neural network while concurrently diminishing computational demands. Conversely, the Focus module processes the image by slicing it, amplifying the input channel fourfold, and then undergoing a convolution. This sequence yields a downsampled feature map, optimizing computational efficiency and bolstering processing speed through effective downsampling. The Neck module integrates both the Feature Pyramid Network (FPN) and the Path Aggregation Network (PAN). The FPN structure facilitates the transfer and fusion of advanced feature data via upsampling, whereas the PAN directs potent positioning features from lower to upper layers. The synergistic interplay of these structures orchestrates feature fusion across various backbone layers to their corresponding detection layers.

#### 2.3.2. DeepLabv3+ Network Model

While the object detection algorithm effectively localizes the sow’s vulva region, it fails to provide a detailed segmentation due to the irregularities inherent in the sow’s vulva shape. Some researchers have sought to integrate object detection algorithms with morphological techniques for thermal window region segmentation. However, this approach requires the manual design of the feature extractor [13], and its segmentation robustness remains questionable given the variance in sow vulva shapes. To enhance segmentation precision, this study incorporated the DeepLabv3+ semantic segmentation network subsequent to the YOLOv5 object detection framework, targeting the intricate segmentation of the sow’s vulva.

With advancements in deep learning technology, semantic segmentation methods based on convolutional neural networks (CNN) exhibited widespread applications, from livestock and poultry skeletal image segmentation to organ imaging. Prominent deep learning approaches for semantic segmentation encompass the fully convolutional network (FCN), U-Net, and the DeepLab series, which include DeepLabv1, DeepLabv2, DeepLabv3, and DeepLabv3+ [14,15,16]. In comparison to FCN and U-Net, DeepLabv3+ leverages the atrous spatial pyramid pooling (ASPP) to expand the receptive field without altering resolution, concurrently facilitating the fusion of multi-layered features. Such an approach adeptly addresses multi-scale segmentation challenges [17]. Further, DeepLabv3+ incorporates a decoder module, enhancing the segmentation outcome by compensating for boundary loss [18]. However, this addition increases model intricacy, leading to protracted training and inference durations. Balancing segmentation precision with speed, this study selects the DeepLabv3+ semantic segmentation network as its foundational structure. However, it substitutes the original Xception feature extraction network with the more streamlined MobileNetV2, thereby amplifying segmentation velocity by curtailing model complexity. To preserve segmentation accuracy, the CBAM module is integrated into the network, enhancing its feature extraction prowess [19].

**Figure 7:**
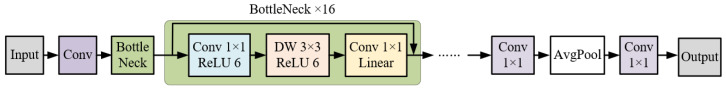
Structure of MobileNetV2 model.

MobileNetV2 stands as a streamlined model, optimized to elevate mobile model performance. It employs depth separable convolution, wherein a standard 3 × 3 convolution is decomposed into a 3 × 3 depthwise convolution (DW) and a 1 × 1 pointwise convolution (PW) [20]. Through such convolutional substitution, MobileNetV2 achieves reduced complexity and accelerated training, without compromising model efficacy. To amplify the feature extraction efficiency of DW convolution, the Bottle-Neck layer incorporates inverted residuals as its core structure. Moreover, to circumvent the potential feature loss attributed to the rectified linear unit (ReLU), the terminal 1×1 convolutional layer within the Bottle-Neck layer deploys a linear activation function, eschewing the ReLU activation. The comprehensive architecture of the MobileNetV2 network is depicted in Figure 7.

#### 2.3.3. CBAM Module

**Figure 8:**
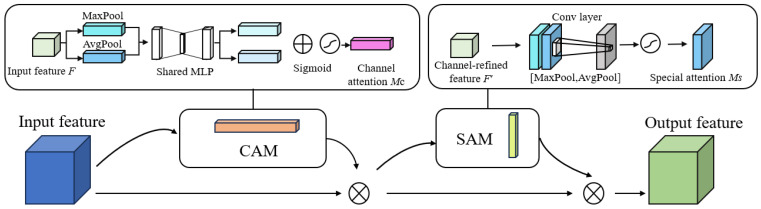
CBAM structure diagram.

The diminutive dimensions of the sow’s vulva region, combined with its subtle edge features, can lead to the extraction of extraneous features during model training, thereby compromising the model’s performance. To address this issue, this study integrates a CBAM module into the network. This aims to enhance the localization and segmentation accuracy for the sow’s vulva region, while amplifying the model’s sensitivity to vital edges and semantic details. The CBAM module is bifurcated into two sub-modules: the Channel Attention Module (CAM) and the Spatial Attention Module (SAM). While SAM is designed to extract the predominant feature information from the input image, CAM assigns weights based on the significance of each feature channel [21]. Figure 8 delineates the architecture of the CBAM module.

In the Channel Attention Module (CAM), both average pooling and maximum pooling are utilized on the input images. Subsequently, a channel attention map is produced through a multi-layer perceptron (MLP). The attention score for each channel is then calculated by summing the elements. The computational equation is delineated below.

(1) $$M_{C}(F)=\sigma (MLP(\mathrm{Avgpool}(F)))+\sigma (MLP(Maxpool(F)))$$

Let *M*_c_(*F*) represent the CAM. Utilizing the sigmoid activation function, denoted as σ, and the MLP, the CAM is computed. Here, AvgPool(*F*) and MaxPool(*F*) signify average pooling and maximum pooling, respectively.

Contrary to the CAM module, the SAM module aims to discern object location information. It conducts both average and maximum pooling operations along the channel axis and subsequently generates a spatial attention map via the convolutional layer. The corresponding mathematical expression is presented below:

(2) $$M_{s}(F^{\prime })=\sigma (f^{7\times 7}[(Avgpool(F^{\prime });Maxpool(F^{\prime })]))$$

where *M_s_* (*F*′) is the SAM; *F*′ represents the feature map generated by the CAM; and *f* ^7×7^ represents the 7 × 7 convolutional layer.

### 2.4. Model Construction for Prediction of Sow Temperature

Recent studies indicate that the accuracy of sow temperature monitoring using thermal infrared imaging is significantly influenced by environmental factors [22]. The prediction error associated with sow temperature can be mitigated by developing a model that predicts sow rectal temperature based on vulval infrared temperature in conjunction with environmental parameters [23]. Concurrently, with the advancement of artificial intelligence (AI), machine learning emerges as a potent tool adept at addressing intricate prediction challenges posed by high-dimensional and large-scale data. Specifically, random forest (RF), an ensemble-based machine learning algorithm, is proficient in delineating intricate relationships between input features and outputs. It is applied in various domains, including sow behavior classification, early disease detection, production capacity forecasting, and pork quality assessment [24,25,26].

**Figure 9:**
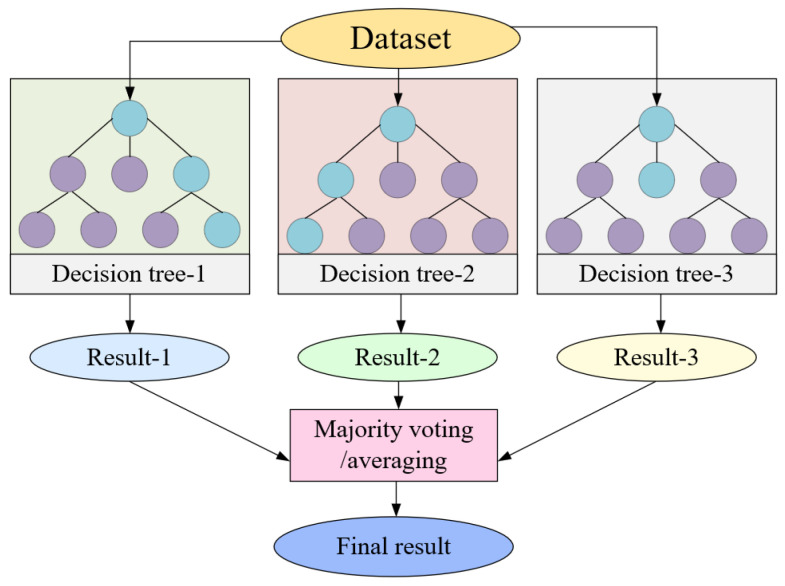
Random forest flowchart.

RF, or random forest, is an ensemble learning algorithm comprising multiple decision trees. It formulates numerous training datasets through random sampling and independently constructs each decision tree. Subsequent to this, it either votes or averages the prediction results to yield regression outcomes. Benefiting from the variance and diversity among its decision trees, RF exhibits notable robustness and generalization capabilities. The fundamental principle of RF is illustrated in Figure 9.

**Figure 10:**
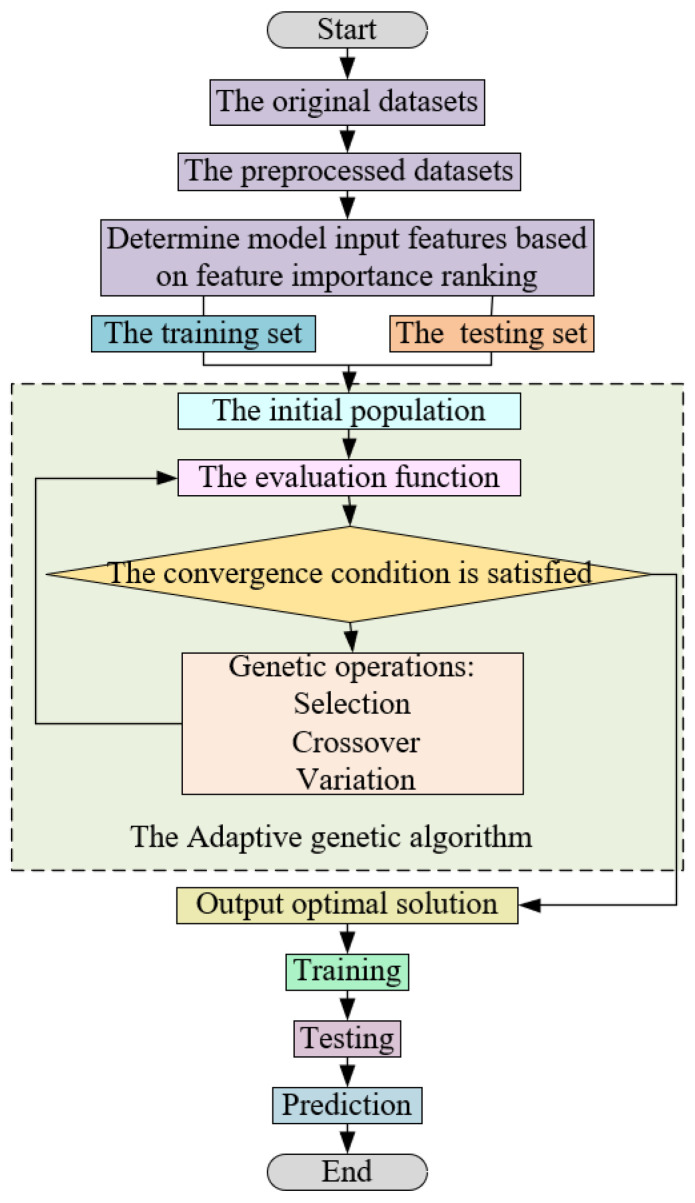
Flow chart of random forest regression model based on adaptive genetic algorithm.

In the gathered IR data, outliers emerge due to factors such as equipment limitations and the activity of the sow. These outliers can compromise the predictive capacity of the model. To tackle this challenge, this study introduces an adaptive genetic algorithm-based random forest method, termed AGA-RF. The procedural flow of the proposed model is depicted in Figure 10. Within this approach, dominant individuals with a high adaptability are subjected to a greater probability of crossover, thereby augmenting the chances of inheriting dominant genes by the next generation, aligning closely with genetic evolution principles. Conversely, individuals with low fitness experience heightened the probability of mutation, facilitating the emergence of new dominant individuals through this operation and preventing the algorithm from becoming trapped in a local optimum. By adaptively modulating crossover and mutation probabilities during genetic operations, the early convergence phenomenon in the genetic algorithm is mitigated. The crossover probability, *P*_c_, and the mutation probability, *P*_m_, in the genetic operation can be defined as follows:

(3) $$P_{c}=\{\begin{array}{l} \{\frac{k_{1}(f_{\max}-f^{\prime })}{\left(f_{\max}-\bar{f}\right)}\;f^{\prime }\geq \bar{f}\\ k_{2}\;f^{\prime }<\bar{f} \end{array}$$

Let *k*_1_ and *k*_2_ be constants within the interval [0, 1]. If *f*_max_ represents the maximum fitness in the current population, *f*′ signifies the maximum fitness value of the two individuals undergoing the crossover operation, and *f*_avg_ denotes the average fitness of the population. Consequently, adopting a lower crossover rate for high-fitness individuals aids in preserving superior genes, while a higher crossover rate for low-fitness individuals facilitates the removal of inferior genes.

(4) $$P_{m}=\{\begin{array}{l} \{\frac{k_{3}(f_{\max}-f)}{\left(f_{\max}-\bar{f}\right)}\;f\geq \bar{f}\\ k_{4}\;f<\bar{f} \end{array}$$

*f* represents the fitness of the individual undergoing the mutation operation, and *k*_3_ and *k*_4_ are constants within the interval [0, 1]. Consequently, adopting a lower mutation rate for high-fitness individuals aids in preserving superior genes. Conversely, a higher mutation rate for low-fitness individuals facilitates the removal of inferior genes.

### 2.5. Model Evaluation Metrics

Model evaluation was bifurcated into two distinct sections. The first section focused on evaluating the performance of the vulval region segmentation model, utilizing metrics such as intersection over union (IoU) and segmentation speed. The second section was dedicated to assessing the sow temperature prediction model with evaluation metrics including mean squared error (MSE), mean absolute error (MAE), and coefficient of determination (R^2^).

(5) $$\mathrm{IoU}=\frac{\left|D\cap M\right|}{\left|D\cup M\right|}$$

where D and M, correspondingly, represent the segmentation result and the ground truth.

(6) $$MSE=\frac{1}{n}\sum {}_{i=1}^{n}{\left(T_{i}-{\overset{⌢}{T}}_{i}\right)}^{2}$$

(7) $$MAE=\frac{1}{n}\sum {}_{i=1}^{n}(T_{i}-{\overset{⌢}{T}}_{i})$$

(8) $$R^{2}=1-\frac{\sum {}_{i=1}^{n}{\left(T_{i}-\overset{⌢}{T}\right)}^{2}}{\sum {}_{i=1}^{n}{\left(T_{i}-\bar{T}\right)}^{2}}$$

where *n* is the number of rectal temperatures in the test set, *T_i_* is the true value of the *i*-th rectal temperature in the test set and is the predicted value of the *i*-th rectal temperature in the test set, and $\overset{⌢}{T_{i}}$ is the average of the actual rectal temperatures.

## 3. Results

### 3.1. Segmentation Model Analysis

#### 3.1.1. Model Training

The model was dependent on the PyTorch 1.7.1 deep learning library, which conformed to the 11th Intel^®^ CoreTM i7-11700k@2.50 HZ×16 (manufactured by Intel Corp., Santa Clara, CA, USA); the graphics card was NVIDIA GTX3090 (manufactured by NVIDIA Corp., Santa Clara, CA, USA); the graphics memory was 24G; and the 64-bit Ubuntu 20.04.1LTS operating system was configured with Python 3.8, Cuda11.4, and OpenCv4.5.1.

**Figure 11:**
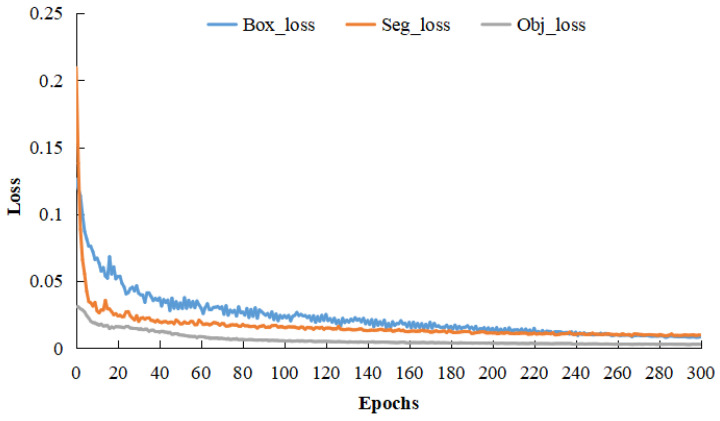
Loss curve.

Upon finalizing the network architecture, the training dataset was inputted for model optimization. Prior to the training process, various network parameters were initialized. The epoch count was set at 300, with a batch size of 24. An initial learning rate of 0.007 was established, which diminished after using cosine annealing. The model was saved at the conclusion of each epoch, with the optimal model chosen for vulvar region segmentation. Figure 11 depicts the trends of box loss, segmentation loss, and object loss across the 300 training epochs. As illustrated in Figure 11, the model’s loss value gradually reduces and stabilizes with increasing epochs. In the final 50 epochs, each loss value remains below 0.01, suggesting a model convergence.

#### 3.1.2. Model Prediction Tests

**Figure 12:**
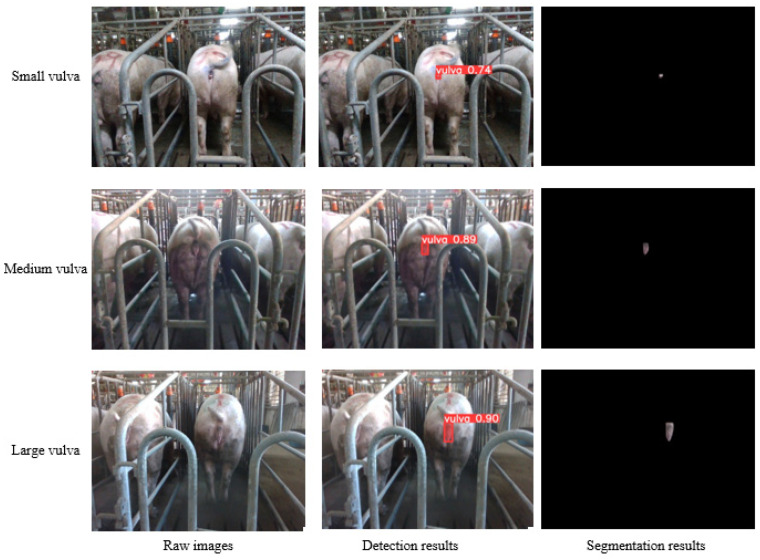
Image segmentation results for different sow vulva sizes.

The vulval size among sows exhibits variability attributable to genetic influences, hormonal levels, and nutritional status. For the appraisal of the segmentation model’s efficacy, the present study opts to analyze 200 images, and each of three distinct vulva sizes—large, medium, and small—are categorized based on dimensions such as length, width, and area. Figure 12 delineates the detection and segmentation outcomes for images, each representative of diverse sow vulva sizes. As manifested in Figure 12, the methodology introduced herein not only precisely identifies the sow’s vulva region amid a complex background but also accurately segments the more diminutive vulva regions. In summation, the presented approach demonstrates a proficient segmentation across varying sow vulva dimensions.

**Figure 13:**
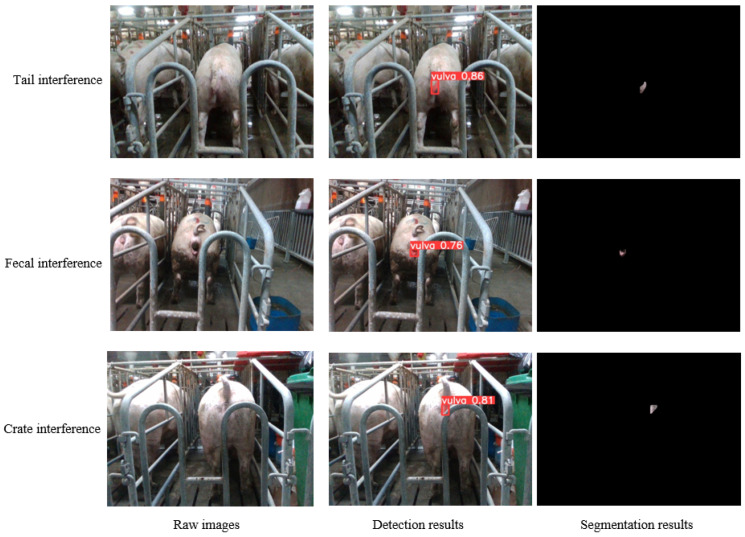
Segmentation results of sow vulva images with different interferents.

In practical sow mating barn scenarios, the image segmentation of the sow vulva area encounters interference from various elements, including the tail, feces, crate, and additional factors. To assess the model’s robustness in navigating diverse interferences, 200 images were selected for each of the three prevalent interferents for examination, with selected test results exhibited in Figure 13. Evident from Figure 13, the method introduced herein adeptly discerns the boundaries between the vulva and other objects, demonstrating a notable robustness against irregular occlusions.

#### 3.1.3. Comparison of Different Segmentation Algorithms

In an extended effort to appraise the segmentation performance of the model, this work juxtaposes the proposed methodology with prevailing segmentation algorithms, such as FCN, U-Net, Mask R-CNN, and You Only Look At CoefficienTs (YOLACT), as detailed in references [27,28]. Table 1 presents the test results obtained from various algorithms. Excluding the present method, the algorithm exhibiting the highest Intersection over Union (IoU) is the Criss-Cross Network (CCNet). CCNet incorporates a criss-cross attention module for each pixel, amassing contextual information from all pixels along its cross-path, thereby enhancing segmentation precision while also elevating the model’s inference time. Apart from the method under discussion, YOLACT records the swiftest segmentation velocity among the compared algorithms by utilizing lower-resolution feature maps to expedite segmentation. While this approach mitigates computational and memory expenditure and accelerates image segmentation, the diminished feature resolution potentially incurs a loss of detail during segmentation, subsequently impacting segmentation accuracy. The model under consideration adeptly curtails computational demands, ensuring segmentation accuracy through an efficient and lightweight network structure, thereby endorsing both exemplary real-time performance and accuracy.

### 3.2. Analysis of Temperature Prediction Models for Sows

#### 3.2.1. Sow Temperature Prediction Tests

**Figure 14:**
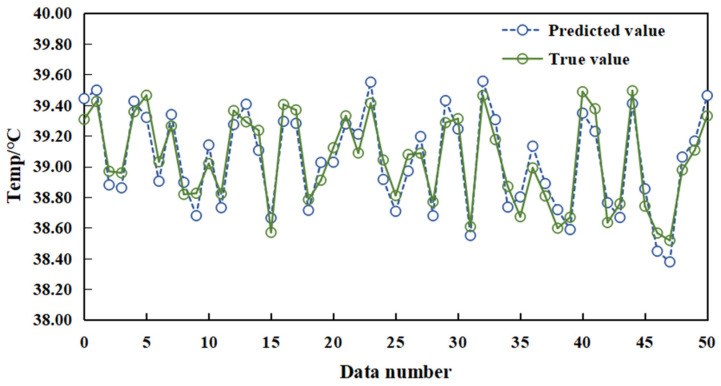
Comparison of real and predicted values of the algorithm proposed in this paper.

Environmental data, thermal infrared temperature metrics, and rectal temperature readings of sows were normalized and utilized as input for network training, facilitating the construction of the sow temperature prediction model. The evaluative process for the performance of the prediction model is executed by employing the formulated temperature prediction model to analyze the data. The subsequent comparison of predictive results with actual rectal temperatures is depicted in Figure 14. Evident from Figure 14, the forecasted temperature values demonstrate proximity to the actual temperature readings. The proposed method exhibits an MSE, MAE, and R^2^ of 0.114 °C, 0.191 °C, and 0.845, respectively, indicating the proficient predictive capabilities of the model.

#### 3.2.2. Comparison of Different Prediction Algorithms

**Figure 15:**
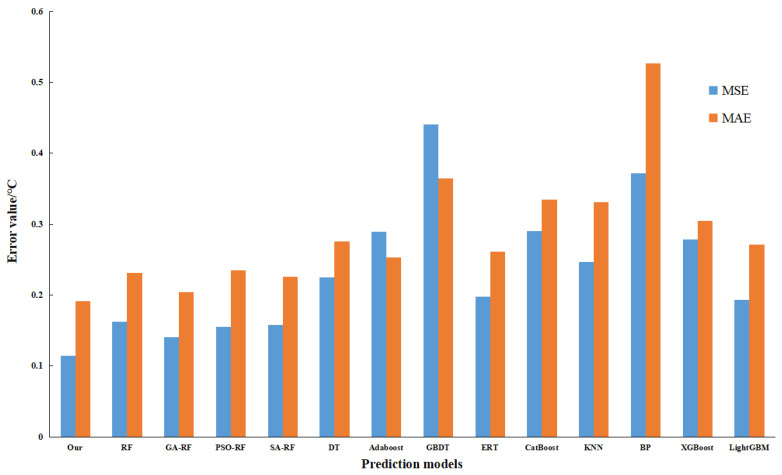
Comparison of MSE and MAE for different algorithms.

**Figure 16:**
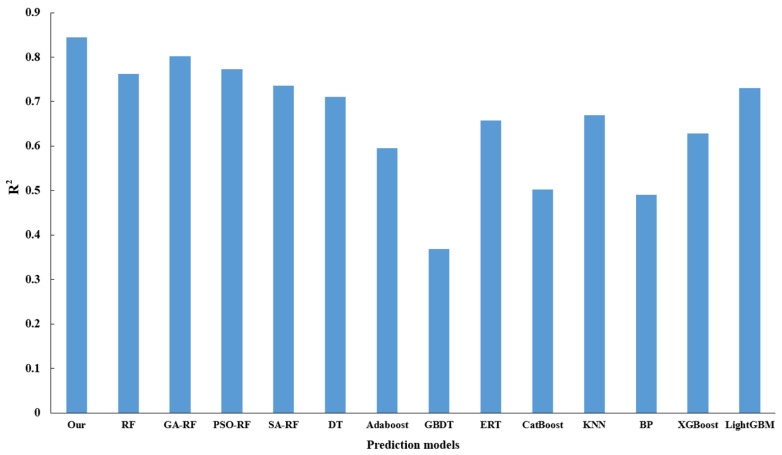
Comparison of R^2^ values for different algorithms.

To assess the optimization efficacy of the model, the present study engages in a comparative analysis involving RF, genetic algorithm augmented with random forest (GA-RF), and the proposed algorithm, with the latter demonstrating superiority across all evaluated indicators, thus affirming that an adaptive genetic algorithm can enhance the predictive capacity of RF. Additionally, an exploration into the optimization performance of varied parameter optimization methodologies for the random forest, underpinned by the Simulated Annealing (SA) algorithm, reveals that the Particle Swarm Optimization (PSO) algorithm fails to effectively optimize the random forest algorithm. In a concluding evaluation, centered around the performance of disparate regression prediction algorithms, a comparative study is conducted involving mainstream regression algorithms, including Decision Tree (DT), Adaptive Boosting (AdaBoost), Gradient Boosting Decision Tree (GBDT), Extremely Randomized Tree (ERT), Categorical Boosting (Catboost), k-Nearest Neighbors (KNN), Back Propagation Neural Network (BP), Extreme Gradient Boosting (XGBoost), and Light Gradient Boosting Machine (LightGBM). The ensuing results affirm that the predictive method proposed herein proves more effective. Figure 15 delineates the MSE and MAE for the varied models, while Figure 16 provides a display of R^2^ values for the assorted models.

## 4. Discussion

### 4.1. Comparison of Different Methods of Temperature Monitoring

To underscore the preeminence of the temperature measurement method introduced in this study, a comparative analysis with preceding studies is conducted. Reference [29] delineates a temperature measurement method for sows, predicated on an implantable thermometry device, whereby the subcutaneous temperature of sows is acquired in real time via a chip implanted beneath the skin on the neck of the sows [29]. Although this method boasts a high-precision temperature measurement, it necessitates individual anesthesia of the sows, posing a challenge for widespread application in large-scale farms. Conversely, reference [30] employs a vaginal probe to record sow temperature. While eschewing the requirement for surgical implantation, this method remains susceptible to disturbances emanating from the sows’ daily activities and is encumbered by a limited device lifespan [30]. Both methods, being contingent upon temperature measurement tests conducted on a modest number of sows, accrue significant costs in terms of time, labor, and equipment, particularly in the management of extensive sow production. In contrast, the method propounded in this paper is non-invasive, efficient, and obviates cross-contamination.

Among non-contact temperature measurement methodologies, reference [31] introduces a wireless sensor network-based strategy for monitoring sow temperature [31]. This technique employs an infrared temperature sensor to ascertain the temperature at the sow’s hip and invokes ambient temperature for the correction of the infrared temperature readings. While this method is financially accessible and simple to install, the infrared temperature sensor encounters challenges in accurately pinpointing the sow’s thermal window area, leading to significant measurement discrepancies. Moreover, the intricate environment of the mating barn precipitates an unstable data transmission. Reference [32] suggests a temperature measurement approach anchored in ultrasonic technology, which is noteworthy for its dynamic temperature measurement and swift response. However, this methodology exhibits substantial temperature measurement errors and fails to accurately trace the temperature fluctuations of sows [32]. In comparison to the aforementioned temperature measurement strategies, the method presented herein offers an enhanced accuracy in temperature measurement, satisfactorily fulfilling the requirements for temperature measurement in large-scale sow farms.

In exploring infrared thermography-based temperature measurement methods for sows, reference [33] utilized infrared thermography to discern temperature variations at the sow’s ear root, swiftly acquiring temperatures in the sows’ thermal window area. However, this method did not accommodate for temperature losses attributable to environmental disturbances and equipment-related variables [33]. Conversely, reference [34] proposed a method to detect ear root feature regions in thermal infrared images of pigs, based on an enhanced Otsu algorithm. Nonetheless, this method is only applicable for pig images with complete ear root features, and the algorithmic complexity remains high [34]. Distinguished from the aforementioned methods, the present method employs environmental parameters to counterbalance the temperature losses from environmental disturbances and accurately segments the incomplete thermal window region. Reference [35] introduced a method to extract the vulva region of sows, based on the improved YOLOv5s object detection algorithm [35]. In comparison, the method proposed herein can precisely delineate the sow vulva boundary, accommodate variations of sow vulva across different sizes, and enhance the accuracy and reliability of thermal infrared temperature measurement.

### 4.2. Limitations and Future Work

Despite the reliability and accuracy of the implemented method, several limitations persist. Initially, challenges arise in the data collection system’s ability to precisely capture the vulva area of the sow, particularly when it is entirely obscured by a bar or when the sow adopts a sitting or lying position.

Future work will contemplate employing multiple thermal infrared data acquisition modules, strategically positioned at various angles, to capture sow images, thereby mitigating the influence of barn shading on temperature measurements. Moreover, the actual sow farm environment encapsulates more complexity; environmental factors, such as temperature and humidity within the barn, may fluctuate according to season, time of day, and management practices. Forward-looking endeavors will seek to expand the dataset further to enhance the model’s generalization capability.

## 5. Conclusions

To reduce labor and material resources required for traditional sow temperature monitoring, the present study introduces an infrared thermography-based method. This method encompasses the development of a segmenter, utilizing YOLOv5s and DeepLabv3+, to facilitate the segmentation of the sow’s vulva region. In pursuit of an enhanced accuracy in the localization and segmentation of this region, Convolutional Block Attention Module (CBAM) was integrated into the segmenter. Moreover, to augment the model’s segmentation speed, the MobileNetV2 lightweight backbone network was implemented. The construction of a temperature compensation prediction model, based on the Adaptive Genetic Algorithm-Random Forest (AGA-RF), was established to achieve accurate sow temperature predictions. The sow image segmentation speed achieves 49.26 frames per second (fps), and the mean squared error (MSE) of the temperature prediction is merely 0.114 °C, satisfying the exigencies of sow temperature monitoring in actual production.

Our method can quickly and accurately predict sow temperature, effectively reducing sow stress caused by manual temperature measurement, decreasing sow production costs, and improving sow management efficiency. It merits noting that all data are autonomously collected by the data acquisition system, thereby facilitating the ease of popularization and application in sow production management detection, health screening, among other uses.

## Figures and Tables

**Table 1:** Performance of different segmentation algorithms.

Model	IoU/%	Speed/fps
Ours	91.50	49.26
FCN	88.28	33.81
Mask R-CNN	89.06	11.26
YOLACT	80.86	37.76
U-Net	86.38	17.20
ISANet	89.30	37.78
ANN	88.89	32.84
GCNet	89.33	33.53
EncNet	88.74	36.56
PSANet	88.82	26.22
DANet	87.99	30.97
NonLocalNet	89.35	31.00
UPerNet	88.30	34.72
CCNet	89.52	32.84
PSPNet	89.29	34.38

## Data Availability

Not applicable.
